# PPARγ Downregulation by TGFß in Fibroblast and Impaired Expression and Function in Systemic Sclerosis: A Novel Mechanism for Progressive Fibrogenesis

**DOI:** 10.1371/journal.pone.0013778

**Published:** 2010-11-02

**Authors:** Jun Wei, Asish K. Ghosh, Jennifer L. Sargent, Kazuhiro Komura, Minghua Wu, Qi-Quan Huang, Manu Jain, Michael L. Whitfield, Carol Feghali-Bostwick, John Varga

**Affiliations:** 1 Division of Rheumatology, Northwestern University Feinberg School of Medicine, Chicago, Illinois, United States of America; 2 Division of Respiratory and Critical Care Medicine, Northwestern University Feinberg School of Medicine, Chicago, Illinois, United States of America; 3 Department of Genetics, Dartmouth Medical College, Hanover, New Hampshire, United States of America; 4 Division of Pulmonary, Allergy, and Critical Care Medicine, University of Pittsburgh School of Medicine, Pittsburgh, Pennsylvania, United States of America; Louisiana State University, United States of America

## Abstract

The nuclear orphan receptor peroxisome proliferator-activated receptor-gamma (PPAR-γ) is expressed in multiple cell types in addition to adipocytes. Upon its activation by natural ligands such as fatty acids and eicosanoids, or by synthetic agonists such as rosiglitazone, PPAR-γ regulates adipogenesis, glucose uptake and inflammatory responses. Recent studies establish a novel role for PPAR-γ signaling as an endogenous mechanism for regulating transforming growth factor-ß (TGF-ß)-dependent fibrogenesis. Here, we sought to characterize PPAR-γ function in the prototypic fibrosing disorder systemic sclerosis (SSc), and delineate the factors governing PPAR-γ expression. We report that PPAR-γ levels were markedly diminished in skin and lung biopsies from patients with SSc, and in fibroblasts explanted from the lesional skin. In normal fibroblasts, treatment with TGF-ß resulted in a time- and dose-dependent down-regulation of PPAR-γ expression. Inhibition occurred at the transcriptional level and was mediated via canonical Smad signal transduction. Genome-wide expression profiling of SSc skin biopsies revealed a marked attenuation of PPAR-γ levels and transcriptional activity in a subset of patients with diffuse cutaneous SSc, which was correlated with the presence of a “TGF-ß responsive gene signature” in these biopsies. Together, these results demonstrate that the expression and function of PPAR-γ are impaired in SSc, and reveal the existence of a reciprocal inhibitory cross-talk between TGF-ß activation and PPAR-γ signaling in the context of fibrogenesis. In light of the potent anti-fibrotic effects attributed to PPAR-γ, these observations lead us to propose that excessive TGF-ß activity in SSc accounts for impaired PPAR-γ function, which in turn contributes to unchecked fibroblast activation and progressive fibrosis.

## Introduction

Systemic sclerosis (SSc) is a complex autoimmune disease of unknown etiology associated with early and transient inflammation and vascular injury, followed by progressive fibrosis affecting the skin and multiple internal organs [1]. Fibrosis, the distinguishing pathological hallmark of SSc, is characterized by overproduction of collagen and other extracellular matrix (ECM) components and accumulation of α-smooth muscle actin (ASMA)-positive myofibroblasts, resulting in excessive connective tissue accumulation and matrix remodeling.

Unchecked fibroblast activation in SSc is the result of a complex series of events orchestrated by transforming growth factor-ß (TGF-ß), a multifunctional cytokine that is produced and secreted by most cell types, and also stored in a latent form in the pericellular matrix [2]. TGF-ß signaling is initiated by ligand binding to the type II TGF-ß receptor (TßRII), which leads to activation of the type I TGF-ß receptor (TßRI). Activated TßRI phosphorylates cytoplasmic Smad2 and Smad3, promoting the formation of Smad heterocomplexes and their nuclear accumulation. Within the nucleus, the Smad complex selectively binds to conserved Smad binding element (SBE) DNA sequences, recruits the histone acetyltransferase p300 and other coactivators, and activates or represses the transcription of target genes such as pro-αI (1) collagen (COL1A1) and collagenase-1, respectively [3], [4], [5], [6].

Peroxisome proliferator-activated receptor-gamma (PPAR-γ) is a nuclear orphan receptor and ligand-inducible transcription factor. Initially identified in adipocytes, PPAR-γ was shown to function as a key regulator of normal adipogenesis [7]. During the past decade, it has become apparent that PPAR-γ is widely expressed in mammalian tissues, and regulates not only genes involved in lipid uptake, synthesis and storage, but also glucose metabolism, cell differentiation, survival and proliferation, and immune and inflammatory responses [8], [9]. Naturally-occurring ligands such as 15-deoxy-Δ12, 14-prostaglandin J_2_ (15d-PGJ_2_) and lysophosphatidic acid (LPA), as well as synthetic ligands such as pioglitazone and rosiglitazone, and triterpenoids such as 2-cyano-3,12-dioxooleana-1,9(11)-dien-28-oic acid (CDDO), directly bind to intracellular PPAR-γ and trigger conformational changes that results in recruitment of the transcriptional coactivators steroid receptor coactivator, retinoid X receptor-alpha and p300/CBP [10]. Within the nucleus, the ligand-PPAR-γ complex binds to PPAR response element (PPRE) DNA sequences of target genes, and positively or negatively regulates their transcription [11].

In addition to its well-established impact on adipogenesis and glucose homeostasis, PPAR-γ appears to have an important novel role in the negative regulation of connective tissue biosynthesis, and is increasingly implicated in both physiologic and pathological matrix remodeling [12]. We have shown that natural and synthetic PPAR-γ ligands, while having little effect on basal rates of collagen synthesis and ASMA expression, effectively blocked the stimulation of these fibrotic responses elicited by TGF-ß [13]. The potent anti-fibrotic effects of the PPAR-γ ligands involved the disruption of Smad-dependent intracellular signal transduction via PPAR-γ-mediated transrepression [14]. Subsequent studies using a variety of cell types have confirmed and expanded on these findings [15], [16], [17]. Moreover, treatment with synthetic PPAR-γ ligands was shown to attenuate experimentally-induced hepatic [18], cardiac [19] and kidney [20] fibrosis, and prevent bleomycin-induced scleroderma [21], in vivo. Additionally, the expression of PPAR-γ is markedly diminished in lesional tissues in rodent models of fibrosis and scleroderma [21], [22], [23], as well as in lesional skin from patients with cicatrical (scarring) alopecia [24]. These observations provide compelling support for a physiological role for PPAR-γ in modulating fibroblast activation and matrix remodeling.

In light of the ability of ligand-activated PPAR-γ to disrupt intracellular TGF-ß signal transduction and prevent excessive fibroblast activation, its levels and biological availability in a particular cell or tissue might be one of the critical factors governing the duration and intensity of profibrotic responses. Indeed, we have shown that augmenting cellular PPAR-γ in normal fibroblasts by ectopic overexpression markedly enhanced their sensitivity to PPAR-γ agonists [13]. However, the mechanisms that regulate the cell-type-specific abundance and activity of PPAR-γ are poorly understood. We therefore sought to investigate the regulation of PPAR-γ expression in SSc, and in explanted lung and dermal fibroblasts. We show here that PPAR-γ expression was markedly diminished in skin and lung biopsies from patients with diffuse cutaneous SSc, as well as in unstimulated fibroblasts explanted from the lesional skin. Incubation of normal fibroblasts with TGF-ß induced a time- and dose-dependent suppression of PPAR-γ gene transcription and activity. Inhibition was mediated via the canonical Smad signal transduction pathway. These results reveal a reciprocal functionally antagonistic relationship between ligand-dependent PPAR-γ signaling that blocks Smad-mediated TGF-ß responses, and Smad-mediated TGF-ß signaling which is shown here to suppress the transcription and activity of PPAR-γ. Unchecked TGF-ß signaling in lesional tissue might account for the impaired local PPAR-γ expression and function in patients with dcSSc. Fibroblasts activated via autocrine or paracrine TGF-ß and consequently defective in PPAR-γ function might be unable to properly modulate TGF-ß signaling, resulting in unchecked fibrogenic stimulation that could contribute to persistence and progression of fibrosis in SSc.

## Results

### PPAR-γ expression is reduced in systemic sclerosis

**Figure 1:**
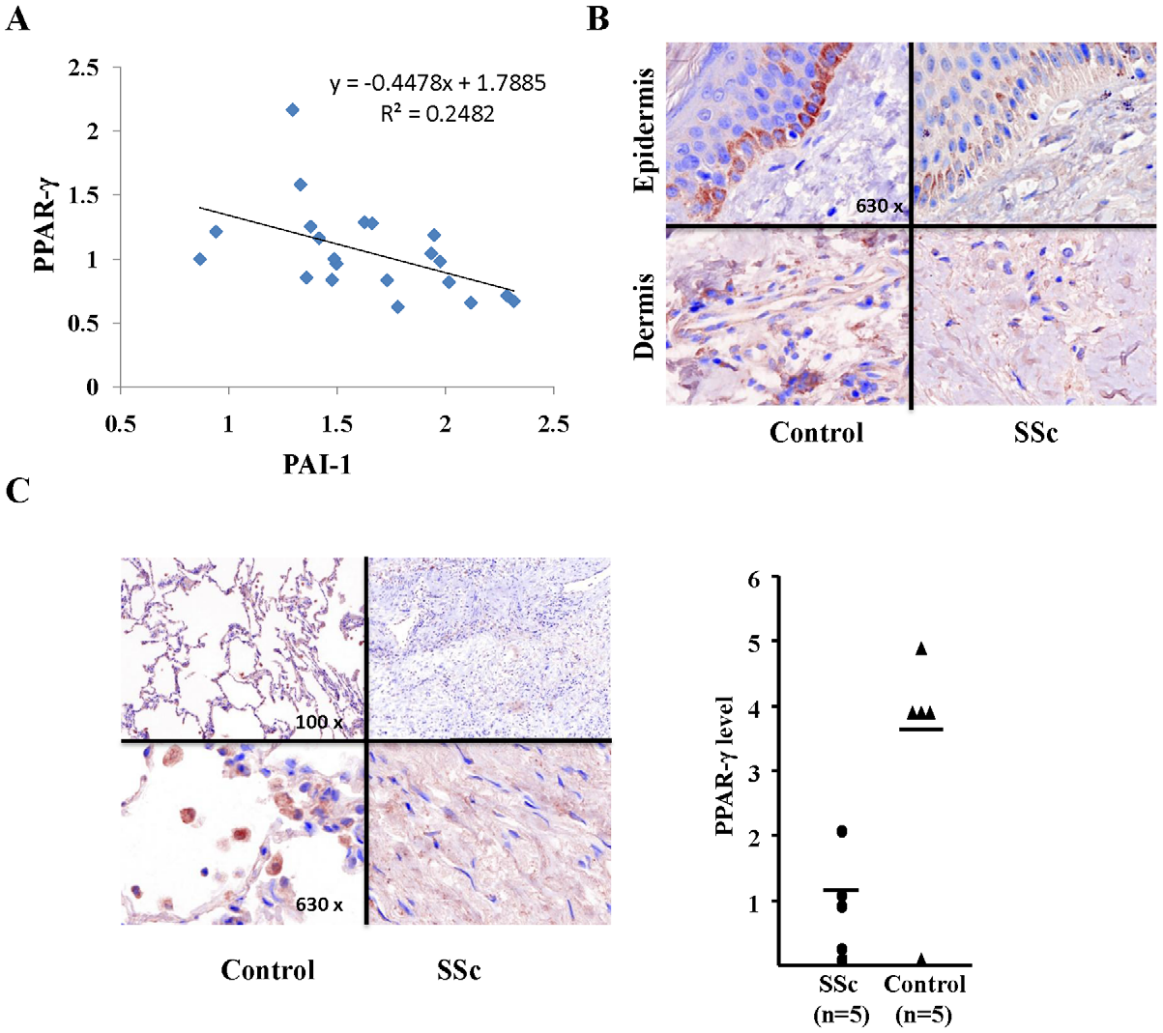
PPAR-γ expression is reduced in SSc. **A.** Expression of PPAR-γ and PAI-1 mRNA was determined by analyzing a previously described skin biopsy microarray dataset from a well-characterized cohort of scleroderma patients (n = 17) [25]. The fold change of mRNA levels was normalized with the average expression level of the samples of whole cohort. Each point represents the relative PPAR-γ and PAI-1 mRNA levels in dcSSc forearm biopsies. **B.** Skin biopsies from patients with dSSc (n = 3) and normal controls (n = 3) were immunostained with antibodies to PPAR-γ. **C.** Lung biopsies from patients with SSc-associated advanced pulmonary fibrosis (n = 5) and non-fibrotic controls (n = 5) were immunostained with antibodies to PPAR-γ. Left panels, representative images. Sections incubated with preimmune serum showed absence of staining. Right panel, expression of PPAR-γ in the biopsies was scored from 0 (no immunostaining) -5 (strong immunostaining). The results shown as symbols represent the means from five separate fields per section for each sample at magnification of 630x, and the horizontal lines represent the means.

In light of the potent anti-fibrotic activities associated with the PPAR-γ pathway, and the demonstration of diminished PPAR-γ expression in various models of fibrosis, we sought to explore the possibility that deregulated fibrogenesis in SSc was accompanied by tissue-specific alterations in PPAR-γ. To examine genome-wide changes in RNA expression, a previously described skin biopsy-derived microarray dataset was interrogated [25]. Analysis of the data showed that levels of PPAR-γ mRNA in the skin were reduced in biopsies from dcSSc patients compared to healthy controls (Fig. 1A). In contrast, plasminogen activator inhibitor-1 (PAI-1), a TGF-ß-regulated gene that is associated with fibrosis and implicated in its pathogenesis, showed increased expression in SSc biopsies [26], [27]. Intriguingly, expression levels of PAI-1 and PPAR-γ mRNA were inversely correlated in these samples (Fig. 1A).

To evaluate the levels of PPAR protein in the skin, biopsies from three patients with SSc and three healthy controls were examined by immunohistochemistry. Low levels of PPAR-γ could be detected in the normal dermis, consistent with prior reports [28]. Detectable PPAR-γ in these skin biopsies was localized mostly to the cells of the epidermis, with scattered dermal cells showing positive immunostaining (Fig. 1B). Lesional skin biopsies from patients with dcSSc consistently showed markedly reduced PPAR-γ immunostaining in the epidermis, and a complete absence of PPAR-γ in the dermis.

Because pulmonary fibrosis commonly complicates SSc, we next examined the expression of PPAR-γ in the lungs. For this purpose, lung biopsies from five SSc patients with advanced pulmonary fibrosis and five individuals with no pulmonary fibrosis were examined by immunohistochemistry. Strong immunostaining was evident in control lungs, with prominent PPAR-γ expression seen in interstitial fibroblasts, alveolar lining cells and macrophages, and displaying a predominantly nuclear distribution (Fig. 1C). In striking contrast, we found that PPAR-γ was virtually undetectable at fibrotic loci in lung biopsies from all five patients with SSc. Immunostaining was specific for PPAR-γ, as substitution of the primary antibody with non-specific mouse IgG resulted in complete absence of staining (not shown).

### Reduced PPAR-γ in explanted SSc fibroblasts

**Figure 2:**
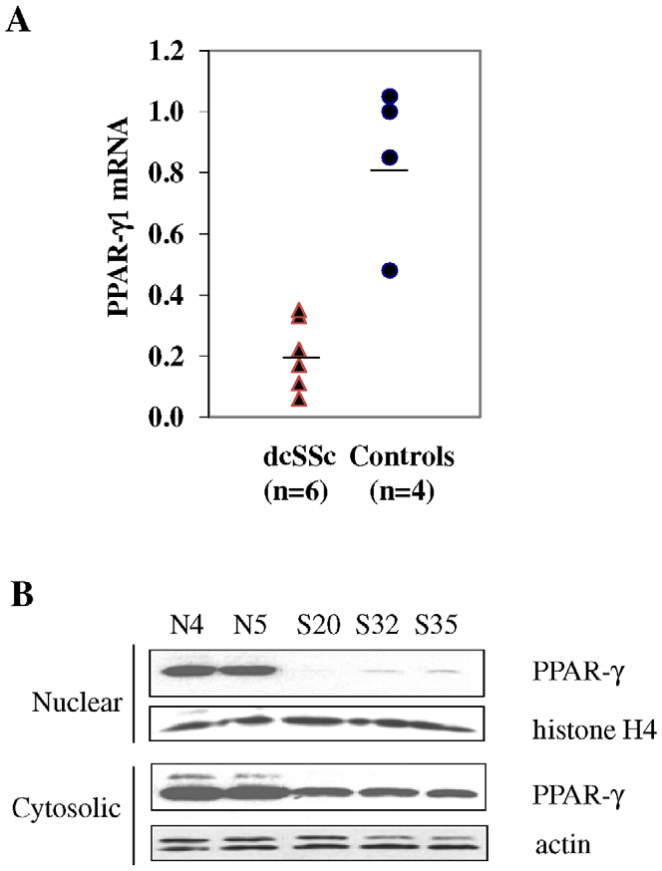
PPAR-γ expression is reduced in explanted SSc skin fibroblasts. Dermal fibroblasts explanted from patients with dcSSc (n = 6) or healthy controls (n = 4) were harvested at confluence. **A.** RNA was subjected to real-time qPCR. The results shown as symbols represent the means of triplicate determinations normalized with ß-actin, and the horizontal lines represent the means. **B.** Nuclear and cytosolic fractions were subjected to Western analysis. Representative immunoblots.

To further characterize PPAR-γ expression in SSc, we examined explanted lesional skin fibroblasts. The clinical characteristics of the patients studied are shown in Table 1. Total RNA was isolated from confluent low-passage SSc and healthy control skin fibroblasts and examined in parallel by real-time qPCR. The results showed substantially diminished levels of PPAR-γ mRNA in lesional fibroblasts from six SSc patients (Fig. 2A). The mean reduction in PPAR-γ mRNA levels was >70% (p = 0.0006) compared to healthy controls. These results were reproducible and consistent over serial passage of the fibroblasts. To compare the cellular abundance of PPAR-γ protein, nuclear fractions were subjected to Western analysis. All three SSc fibroblast lines showed dramatically reduced levels of nuclear PPAR-γ compared to healthy control fibroblasts (Fig. 2B). Taken together, these results demonstrate deficient PPAR-γ mRNA and protein expression in skin and lung biopsies and in explanted dermal fibroblasts from patients with dcSSc.

**Table 1:** Characteristic of patients with SSc.

Subject	Age (years)	Sex	Disease duration* (years)	Sample	SSc subset•
SSc25	29	F	2	Lung	dcSSc
SSc26	54	F	11	Lung	dcSSc
SSc29	64	F	20	Lung	dcSSc
SSc30	63	M	13	Lung	dcSSc
SSc31	43	F	<1	Lung	dcSSc
S20	61	M	1	Skin	dcSSc
S26	52	F	2	Skin	dcSSc
S30	47	F	4	Skin	dcSSc
S32	40	F	5	Skin	dcSSc
S35	58	F	3	Skin	dcSSc
SK	45	M	2	Skin	dcSSc

*From first non-Raynaud syndrome.

• dcSSc, diffuse cutaneous SSc.

### TGF-ß suppresses PPAR-γ expression in normal fibroblasts

In light of the central role that TGF-ß plays in mediating the pathogenesis of fibrosis, we sought to examine the possibility that it might modulate the expression of PPAR-γ, and that activated TGF-ß signaling could account for impaired PPAR-γ expression seen in SSc. Previous studies have described the effects of TGF-ß on gene expression in normal dermal fibroblasts using DNA microarray analysis [29]. Analysis of the data showed that TGF-ß treatment of two independent lines of dermal fibroblasts was associated with a time-dependent decrease in PPAR-γ mRNA expression (data not shown) that was accompanied by a similar inhibition of MMP-1 and Smad3, as we had described previously [3], [6]. This decrease was mirrored by an increase in the expression of fibrosis-related genes such as ASMA, SERPINE1, CTGF and COMP1. In separate experiments, normal lung fibroblasts incubated with TGF-ß showed an identical decrease in PPAR-γ mRNA expression (data not shown).

**Figure 3:**
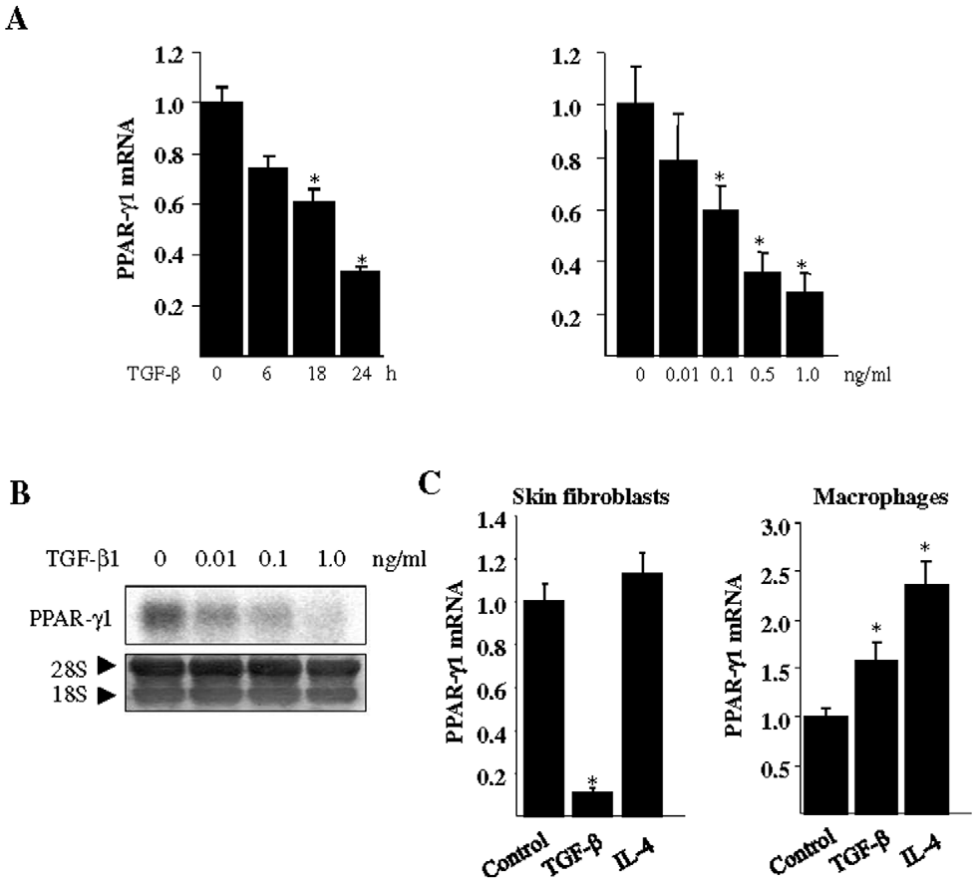
TGF-ß suppresses PPAR-γ mRNA expression in fibroblasts. **A.** Confluent fibroblasts were incubated with 10 ng/ml TGF-ß for indicated period (left panel), or for 24 h with indicated concentrations of TGF-ß (right panel). RNA expression was examined by real-time qPCR. The results, normalized with ß-actin mRNA, represent the means ± SD of triplicate determinations. *p<0.05. **B.** Total RNA was isolated following 24 h incubation with TGF-ß, and subjected to Northern analysis. **C.** Fibroblasts and monocyte-derived macrophages were incubated with TGF-ß or IL-4 (both at 10 ng/ml) in parallel for 24 h, and RNA was examined by real-time qPCR. Results represent the means ± SD of triplicate determinations. * p<0.05.

To further explore the regulation of PPAR-γ mRNA expression, confluent normal dermal fibroblasts were incubated with 10 ng/ml TGF-ß for various periods. Under the experimental conditions employed, there was no detectable cell toxicity due to TGF-β (data not shown). The results of real-time qPCR demonstrated a progressive time- and dose-dependent decrease in the levels of PPAR-γ1 and PPAR-γ2 mRNA, with a maximal >70% inhibition after 24 h (Fig. 3A and data not shown). Essentially identical results were obtained when normal lung fibroblasts were incubated with TGF-ß (data not shown). Suppression of PPAR-γ mRNA in TGF-ß-treated fibroblasts was further confirmed by Northern analysis (Fig. 3B). Remarkably, we noted that the negative regulation of PPAR-γ by TGF-ß was cell type-specific. For instance, in sharp contrast to fibroblasts, incubation of monocyte-derived macrophages with TGF-ß caused stimulation of both PPAR-γ1 and PPAR-γ2 mRNA (Fig. 3C and data not shown). Furthermore, we found that the Th2 cytokine IL-4 stimulated PPAR-γ mRNA in macrophages, whereas it had no effect in fibroblasts.

**Figure 4:**
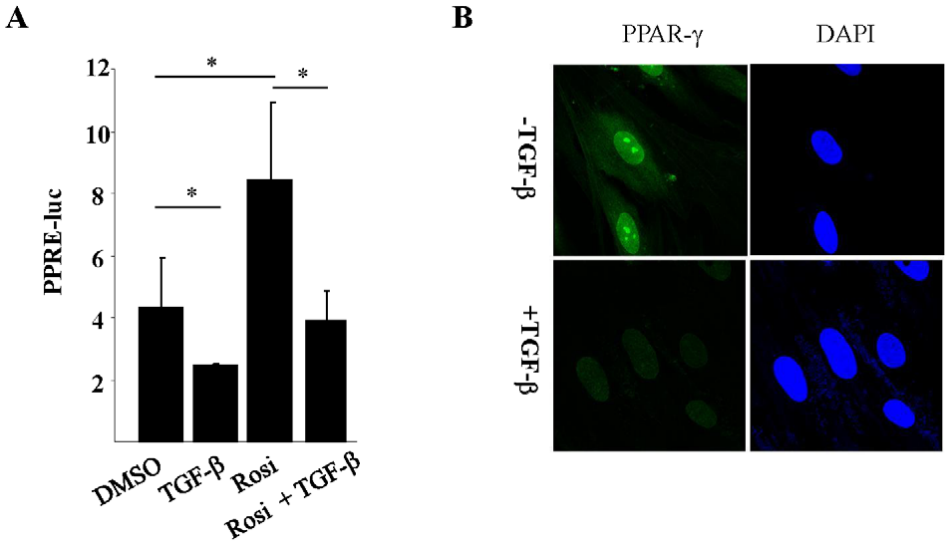
TGF-ß suppresses PPAR-γ expression in fibroblasts. **A**. Confluent normal dermal fibroblasts were transiently transfected with PPRE-luc for 18 h. Cultures were preincubated with rosiglitazone (10 µM) for 60 min, followed by TGF-ß for a further 48 h. Whole cell lysates were assayed for their luciferase activities. The results, normalized with Renilla luciferase activity, represent the means ± SD of triplicate determinations. * p<0.05. **B**. Following incubation with TGF-ß for 48 h, fibroblasts were immunostained using specific antibodies against PPAR-γ (green); DAPI blue. Images were taken under Zeiss UV Meta 510 confocal microscope (original magnification ×400).

To evaluate the functional consequences of inhibition by TGF-ß, we examined the regulation PPAR-γ transcriptional activity. Fibroblasts were transfected with PPRE-luc and, 18 h later, rosiglitazone and TGF-ß were added to the cultures. Following a further 48 h incubation, cultures were harvested and whole cell lysates were assayed for their luciferase activities. The results showed that while rosiglitazone induced a >2-fold increase in the activity of the PPRE promoter, TGF-ß significantly attenuated PPRE-driven luc activity both in the presence and absence of rosiglitazone (Fig. 4A). As expected, the cellular levels of PPAR-γ showed a marked reduction at 48 h in TGF-ß-treated fibroblasts (Fig. 4B). These results indicate that inhibition of PPAR-γ gene expression by TGF-ß was accompanied by reduced transcriptional activity both in the absence and presence of PPAR-γ ligand.

### Suppression of PPAR-γ involves TβRI and the canonical Smad signaling pathway

**Figure 5:**
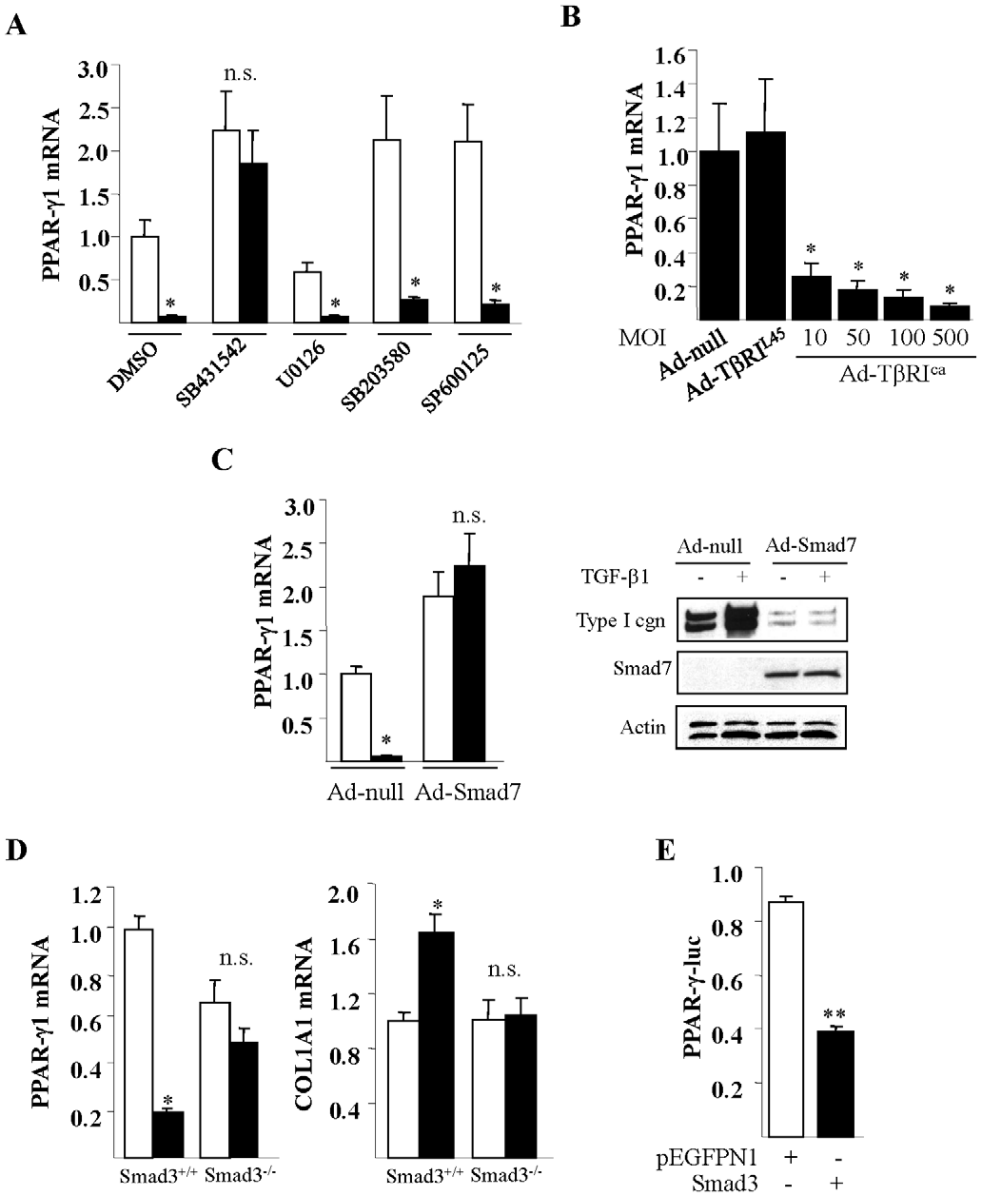
Suppression of PPAR-γ via TßRI and Smad3. **A.** Confluent foreskin fibroblasts were pre-treated with indicated protein kinase inhibitors for 60 min, followed by incubation with 10 ng/ml TGF-ß. Cultures were harvested 24 h later and mRNA levels were analyzed by real-time qPCR. The results, normalized with ß-actin, represent the means ± SD of triplicate determinations. * p<0.05. **B.** Fibroblasts were infected with Ad-null or Ad-TßRI^L45^ (100 MOI) or indicated concentrations of Ad-TßRI^ca^ for 48 h, or **C.** Ad-Smad7 for 24 h (C). RNA subjected to real-time qPCR. The results, normalized with ß-actin, represent the means ± SD of triplicate determinations. * p<0.05. In C, whole cell lysates were subjected to Western analysis. **D.** Confluent cultures of Smad3^−/−^ or Smad3^+/+^ mouse fibroblasts were incubated with 10 ng/ml TGF-ß for 24 h. RNA was subjected to real-time qPCR. The results, normalized with 18S rRNA, represent the means ± SD of triplicate determinations. * p<0.05. **E**. Normal dermal fibroblasts were transiently transfected with PPAR-γ-luc along with Smad3 expression vectors. Following a 24 h incubation, cultures were harvested and whole cell lysates were assayed for their luciferase activities. The results, normalized with Renilla luciferase activity, represent the means ± SD of triplicate determinations. ** p<0.005. In A, C, D, open bars, untreated cultures; closed bars, TGF-ß-treated cultures.

A series of experiments were performed in order to characterize the cellular mechanisms underlying the potent inhibitory effect of TGF-ß on PPAR-γ expression. First, we used selective protein kinase inhibitors to identify intracellular signaling pathways potentially mediating the TGF-ß effect. These studies showed that neither U0126, SB203580 nor SP600125, specific inhibitors of the MEK1/2, p38 mitogen-activated protein kinase and c-jun N-terminal kinase, respectively, abrogated the TGF-ß-induced suppression of PPAR-γ1 and PPAR-γ2 mRNA (Fig. 5A and data not shown). In contrast, the ALK5 inhibitor SB431542, which blocks TßRI-mediated Smad2/3 phosphorylation, almost completely prevented the inhibitory effects. Interestingly, ALK5 blockade by itself caused a >2-fold increase in PPAR-γ mRNA levels, indicating that constitutive TGF-ß signaling causes basal repression of PPAR-γ mRNA in normal fibroblasts. Furthermore, while TGF-ß caused a dose-dependent inhibition of PPAR-γ-luc activity in transiently transfected normal dermal fibroblasts, pretreatment of the cultures with SB431542 completely abrogated this effect (data not shown). These results suggest that TGF-ß-induced inhibition of PPAR-γ occurred at the level of transcription and was mediated via the canonical Smad pathway.

Additional studies were pursued to further explore the role of the Smad signaling in PPAR-γ regulation. First, constitutively active (TßRI^ca^) or non-functional (TßRI^L45^) mutants of TßRI were ectopically expressed in normal fibroblasts. As shown in Fig. 5B, Ad-TßRI^ca^ induced a dose-dependent suppression of PPAR-γ1 mRNA in the absence of exogenous TGF-ß, whereas the non-functional TßRI^L45^ had no effect. Moreover, adenoviral expression of Smad7, an inhibitory SMAD that forms stable association with the TGF-ß receptor complex and prevents phosphorylation of pathway-restricted Smads, blocked the inhibition of PPAR-γ mRNA by TGF-ß (Fig. 5C). As would be expected, Smad7 blocked TGF-ß stimulation of collagen synthesis in infected fibroblasts (Fig. 5C, right panel). Because Smad7 can abrogate both Smad2- and Smad3-dependent responses, we sought to clarify the specific role of cellular Smad3 in PPAR-γ suppression using mouse fibroblasts deficient in Smad3 [30]. The results showed that in contrast to wildtype fibroblasts treated with TGF-ß, in Smad3^−/−^ fibroblasts TGF-ß failed to down-regulate PPAR-γ mRNA (Fig. 5D). As expected, TGF-ß stimulation of Type I collagen gene expression was also lost in Smad3-deficient cells. On the other hand, transient overexpression of Smad3 in normal human dermal fibroblasts was by itself sufficient to repress the activity of the PPAR-γ promoter (Fig. 5E). Taken together, these results firmly establish the critical roles of the canonical Smad signaling pathway involving TßRI and Smad3 as both necessary and sufficient for driving transcriptional suppression of PPAR-γ in response to TGF-ß.

### TGF-ß blocks PPAR-γ–dependent adipogenic differentiation

**Figure 6:**
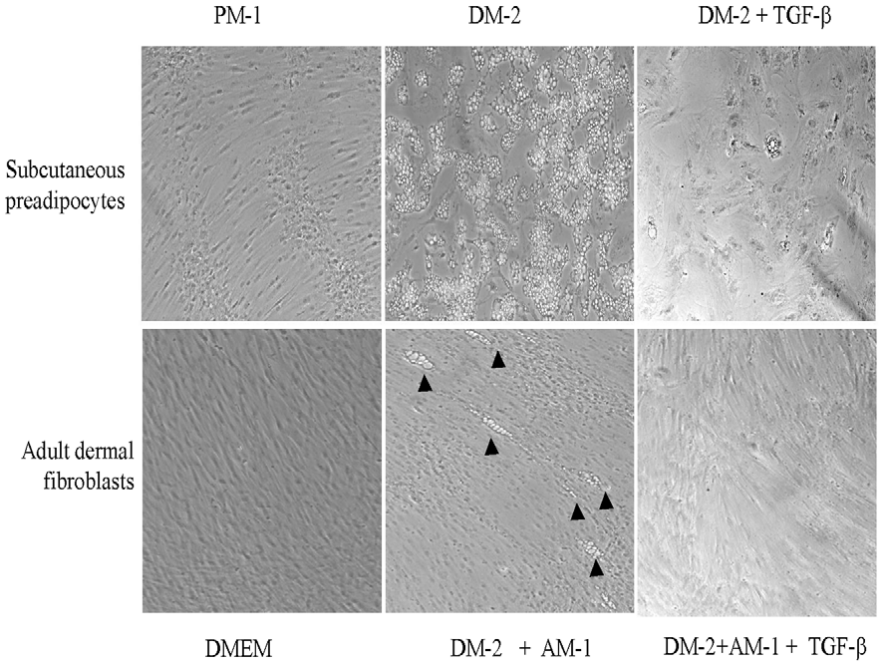
TGF-ß inhibits adipogenic differentiation of mesenchymal cell. Confluent human subcutaneous preadipocytes or adult dermal fibroblasts were induced to undergo adipogenic differentiation in vitro. Subcutaneous preadipocytes were incubated with maintenance media, or differentiation media alone or in the presence of TGF-ß (upper panels). Dermal fibroblasts were incubated with DMEM, or DM-2/AM-1 for three cycles followed by incubation with 10 ng/ml TGF-ß for a further 3 weeks (lower panels), and photographed under a phase contrast microscope. Arrows indicate intracellular accumulation of oil droplets. Original magnification X200.

One of the key physiologic functions of PPAR-γ is the regulation of adipogenesis. Ectopic PPAR-γ is both necessary and sufficient to induce PPAR-γ-dependent adipogenic differentiation in normal mesenchymal cells [7], [31]. The potent inhibitory effect of TGF-ß on the expression and activity of PPAR-γ prompted us to examine the modulation of adipogenesis by TGF-ß. For this purpose, human subcutaneous preadipocytes and normal adult dermal fibroblasts were incubated in adipogenic differentiation media. After three cycles of adipogenic induction, prominent intracellular lipid accumulation was evident in both preadipocytes as well as in dermal fibroblasts (Fig. 6). That these cells underwent adipogenic differentiation was further confirmed by the marked up-regulation of the adipocyte-specific marker FABP4. Inclusion of TGF-ß in the differentiation media resulted in a substantial decrease in PPAR-γ mRNA. Importantly, TGF-ß completely prevented adipogenic differentiation in both subcutaneous preadipocytes and dermal fibroblasts.

### The effect of rosiglitazone in TGF-ß –treated fibroblasts

**Figure 7:**
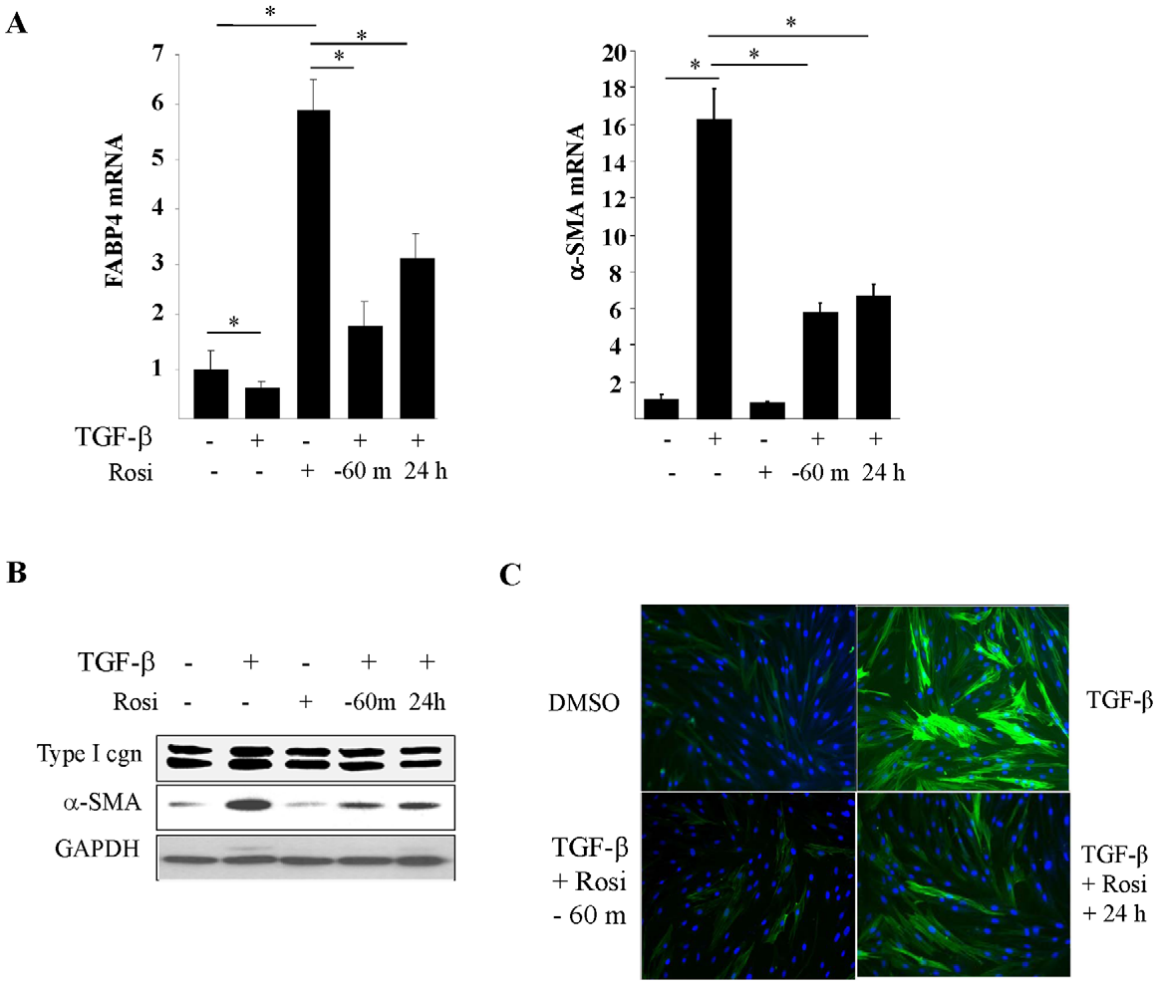
Rosiglitazione abrogates TGF-ß–induced fibroblast activation. Confluent dermal fibroblasts were pretreated with TGF-ß for 24 h, followed by rosiglitazone (10 µM) for further 24 h. **A.** Total RNA was analyzed by real-time qPCR. The results, normalized with 18S, represent the means ± SD of triplicate determinations. * p<0.05. **B.** Whole cell lysates were subjected to Western analysis. **C.** Fibroblasts were fixed and stained with antibodies to α-smooth muscle actin (green color). Nuclei were visualized by DAPI (blue). Original magnification X 200.

By causing a substantial reduction in cellular PPAR-γ abundance, TGF-ß might be expected to attenuate the cell's ability to respond to PPAR-γ ligands. To examine this possibility, the regulation of FABP4, a cardinal endogenous target for activated PPAR-γ was studied. Confluent dermal fibroblasts were incubated with TGF-ß, with rosiglitazone added to the cultures after 24 h. Following a further 24 h incubation, cultures were harvested. As shown in Fig. 7A, in the presence of TGF-ß, rosiglitazone failed to induce full stimulation of FABP4. These results indicate that TGF-ß exposure resulted in partial loss of PPAR-γ–dependent adipogenic effects of rosiglitazone, presumably due to the marked decrease in cellular PPAR-γ abundance induced by exposure to TGF-ß. In contrast, rosiglitazone was capable of partially reducing the stimulation of collagen and ASMA expression when given 24 h following TGF-ß treatment (Fig. 7A–C). These results indicate that TGF-ß markedly attenuated the adipogenic activities of the synthetic PPAR-γ ligand, while only partially attenuating its anti-fibrotic activities, suggesting that the latter might also involve some PPAR-γ-independent mechanisms.

### Reduced expression of PPAR-γ target genes in SSc skin

**Figure 8:**
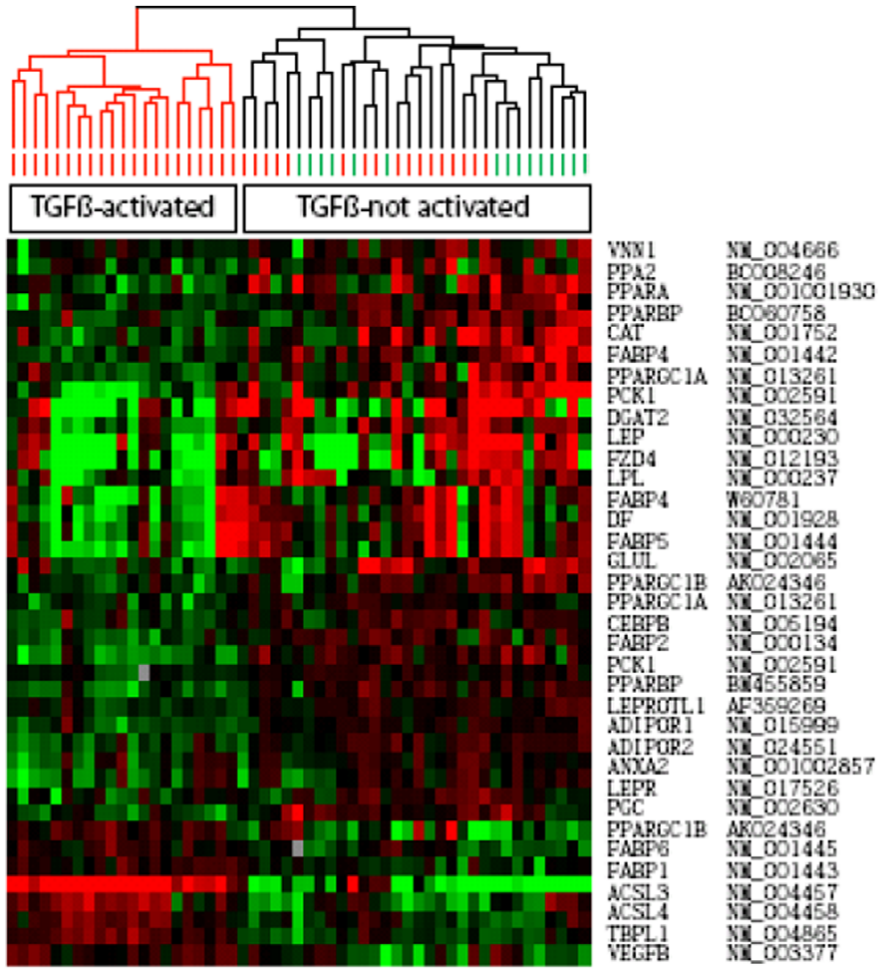
Reduced PPAR-γ target gene expression in SSc skin. Gene expression in lesional skin biopsies was examined by analysis of a previously described microarray dataset based on biopsies from patients with SSc (n = 17, red) and healthy controls (n = 6, green) [29]. Samples were ordered based on their TGF-ß-activated signature (red branches, TGF-ß-activated signature; black branches, TGF-ß not activated). Of forty-one PPAR-γ target genes showing significant differential expression in SSc, thirty-three are down-regulated in samples with TGF-ß-activated gene signature, whereas eight genes show the inverse expression pattern.

In a previous report using microarray-based genome-wide expression profiling of scleroderma skin biopsies, a pattern of gene expression consistent with active TGF-ß signaling was observed in subsets of SSc patients with diffuse cutaneous disease [29]. In light of the potent inhibitory effects of TGF-ß on PPAR-γ demonstrated in our studies, we sought to evaluate the expression of PPAR-γ-regulated genes in the subset of dcSSc patients whose skin biopsies showed a prominent “TGF-ß activated signature”. A list of 87 putative PPAR-γ target genes was compiled from an extensive literature review. The expression of these genes was then examined in a gene expression microarray dataset from a well-characterized cohort of SSc patients [25]. None of these patients were receiving thiazolidinediones at the time of the biopsies. The data, organized by hierarchical clustering based on the expression level of TGF-ß-responsive genes as demonstrated previously [29], are shown as TGF-ß-activated (red) and TGF-ß-not activated (black) clusters. PPAR-γ target genes compiled from literature review showed decreased expression in biopsies from the TGF-ß-activated dcSSc subset compared to the TGF-ß-not activated SSc subset, or to healthy controls (Fig. 8). The significance of differential regulation for each gene was determined using Significance Analysis of Microarrays (SAM). There were forty-one putative PPAR-γ target genes with significant differential expression in the TGF-ß-activated versus TGF-ß-not activated SSc subsets (FDR <0.78%). Thirty-three of these PPAR-γ target genes (80%) showed reduced expression in the TGF-ß-activated group, and elevated expression in the TGF-ß-not-activated group (Fig. 8).

## Discussion

Excessive extracellular matrix accumulation and tissue contraction, the hallmarks of fibrosis, are mediated by fibroblasts and myofibroblasts activated by TGF-ß [1]. Blocking TGF-ß activity therefore represents an appealing approach to fibrosis therapy, and there is intense interest in identifying the intracellular pathways that positively or negatively modulate the fibrotic responses. Recent research findings suggest that PPAR-γ signaling is a novel cell-intrinsic mechanism to negatively regulate profibrotic responses.

PPAR-γ exerts potent antifibrotic effects in vitro and in vivo. We showed previously that PPAR-γ activation by natural or synthetic ligands abrogated collagen synthesis, cell migration and myofibroblast transdifferentiation induced by TGF-ß [21]. While the precise mechanisms underlying abrogation of TGF-ß signaling remain to be fully delineated, we found that PPAR-γ blocked Smad-dependent transcriptional responses without interfering with Smad phosphorylation or nuclear accumulation. Instead, PPAR-γ appears to compete with ligand-inducible transcription factors for binding to limiting p300, squelching Smad/p300 complex formation in TGF-ß-stimulated fibroblasts [14]. Furthermore, in animal models of TGF-ß-dependent fibrosis, rosiglitazone treatment attenuated the development of dermal thickness and collagen accumulation in vivo [21], [32]. Because the biological activities of PPAR-γ ligands depend largely on their interaction with PPAR-γ, the relative levels or biologically availability of PPAR-γ are important factors governing the intensity of ligand-induced cellular responses. Surprisingly, little is known about the expression of PPAR-γ in fibroblasts, and its regulation during inflammation, tissue repair and fibrosis. In the present studies we found that the levels of PPAR-γ protein and mRNA were substantially lower in SSc lesional tissue and explanted skin fibroblasts than in healthy controls. Moreover, genome-wide expression analysis using microarrays demonstrated that SSc skin biopsies with evidence of active TGF-ß signaling had low expression of PPAR-γ-regulated genes, revealing an inverse relationship between TGF-ß activity and PPAR-γ-dependent responses in fibrotic tissues.

We examined the regulation of PPAR-γ by TGF-ß in normal fibroblasts. Despite its importance in determining the intensity of ligand-induced responses, the factors involved in the regulation of PPAR-γ expression are not well understood. Our results demonstrated that TGF-ß caused a potent time- and dose-dependent and cell-type specific down-regulation of PPAR-γ transcription. This inhibitory response was mediated through the canonical Smad signal transduction pathway. A 2.7 kb fragment of the PPAR-γ gene promoter harboring two putative Smad binding elements was sufficient for inhibition by TGF-ß. Taken together with previous findings, the present results indicate that TGF-ß and PPAR-γ are engaged in a mutually antagonistic reciprocal cross-talk, whereby the PPAR-γ pathway abrogates cellular responses induced by TGF-ß, while TGF-ß-Smad inhibits the expression of PPAR-γ and its target genes.

In addition to TGF-ß, IL-13, Wnt, CCN2, as well as hypoxia, have all been shown to inhibit PPAR-γ expression or activity [33], [34], [35], [36], [37]. Particularly interesting is lysophosphatidic acid (LPA), a bioactive lipid that potently inhibits the expression of PPAR-γ in a variety of cell types [38], while also acting as a PPAR-γ agonist [39]. The signaling mechanisms mediating down-regulation of PPAR-γ in response to these cues are not well understood. It is noteworthy that CCN2 and Wnt are known to be up-regulated in the fibrotic cellular milieu in SSc, and chronic tissue hypoxia due to microvascular insufficiency is prominent [1], [40]. Thus, multiple factors implicated in the pathogenesis of fibrosis might contribute to the profound down-regulation of PPAR-γ in SSc. We and others have also observed that PPAR-γ protein and mRNA levels were persistently reduced in explanted SSc fibroblasts [17]. At present, it is unclear whether autonomous PPAR-γ down-regulation represents a cell-inherent abnormality in gene regulation, such as epigenetic modifications at the PPAR-γ promoter, or results from constitutive autocrine TGF-ß stimulation. The latter mechanism is plausible in light of the finding that in normal fibroblasts, constitutive basal TGF-ß signaling maintains tonic suppression of PPAR-γ (Fig. 5A). Epigenetic modification represents an appealing possibility to account for low level of PPAR-γ gene expression, since methylation-mediated silencing of inhibitory transcription factors such as Fli1 or Smad7 has been implicated as a mechanism for autonomous activation of SSc fibroblasts [41],[42]. While we cannot fully exclude the possibility that hypermethylation of the regulatory elements of the PPAR-γ gene result in gene silencing in SSc fibroblasts, in preliminary studies the methylation inhibitor 5-azacytidine failed to normalize PPAR-γ expression (data not shown).

Analysis of microarray data from SSc skin biopsies previously identified a patient subset with a “TGF-ß-responsive gene signature” [25]. This pattern of gene expression was only seen in biopsies from patients with dcSSc, and was not found in biopsies from patients with other forms of scleroderma, or from healthy controls. The “TGF-ß responsive signature” identifies a subset of scleroderma patients with extensive skin involvement and propensity to pulmonary fibrosis [29]. Remarkably we observed that the expression of a majority of differentially regulated PPAR-γ target genes (80%) was diminished in the “TGF-ß responsive gene signature” subset biopsies. These observations reveal an inverse relationship in the tissue expression of TGF-ß-inducible genes and PPAR-γ inducible genes, a result not unexpected in view of our present findings that TGF-ß suppresses PPAR-γ expression and transcriptional activity in fibroblasts, and our previous demonstration that PPAR-γ directly inhibits Smad-mediated TGF-ß responses. The reciprocal relationship between the expressions of these two sets of genes is consistent with a mutually antagonistic cross-talk of the TGF-ß and PPAR-γ pathways. In this context it is remarkable that diminished PPAP-γ expression or function is a recurring theme in human fibrotic conditions and animal models of fibrosis. For example, intracellular PPAR-γ levels were diminished in alveolar macrophages from patients with pulmonary fibrosis [43], and in the fibrotic lesions in cicatrical alopecia [24]. In addition to the association of low PPAR-γ with fibrosis in various human disorders, evidence for a causal relationship comes from animal models. Tissue-specific down-regulation of PPAR-γ, whether due to loss-of-function mutations [44], genetic targeting [45], [46], or overexpression of PPAR-γ inhibitors such as TGF-ß or Wnt [47], [48], is invariably accompanied by exuberant local fibrosis. It is intriguing that in SSc, skin fibrosis is commonly accompanied by a reduction in the subcutaneous fat layer, and in some biopsies, complete adipose tissue atrophy [49]. We speculate that in these cases active TGF-ß signaling in the dermis suppresses PPAR-γ in situ with consequent disruption of adipogenesis, loss of adipocytes and lipoatrophy. These observations highlight the importance of a functional antagonism between profibrotic TGF-ß signaling on the one hand, and the anti-fibrotic PPAR-γ pathway on the other, in the pathogenesis of SSc.

One of the critical mechanisms employed by TGF-β in the promotion of fibrosis is epithelial-mesenchymal transition (EMT) [50]. In this study, we observed reduced levels of epidermal PPAR-γ in SSc skin biopsies, whereas normal skin biopsies showed strong immunostaining in the basal epidermis (Figs 1B). High level of TGF-ß and ASMA expression in the basal epidermis have been reported previously [51]. Because PPAR-γ signaling potently inhibits TGF-β-induced EMT in vitro [52], these observations raise the possibility that an important physiologic role of PPAR-γ in the epidermis is to prevent deregulated EMT, and its loss in SSc may contribute to promotion of EMT and fibrosis.

Since a primary biological function of PPAR-γ is to serve as an intracellular sensor for changes in fatty acid or prostaglandin levels, its abundance and availability are important in determining the intensity of ligand responses. Cellular responses elicited by endogenous ligands, such as adipogenic differentiation of mesenchymal progenitor cells, are dependent on PPAR-γ signaling [31]. We have previously shown that augmenting levels of PPAR-γ by means of ectopic overexpression enhanced the cellular responsiveness to a PPAR-γ ligand [13], [53]. In light of the potent down-regulation of PPAR-γ observed in TGF-ß-treated cells, we anticipated that TGF-ß might reduce cellular sensitivity to PPAR-γ ligands. We found however that fibroblast activation was blunted by rosiglitazone even when it was added to the cultures 24 h after TGF-ß. Therefore, synthetic PPAR-γ ligands are capable of abrogating profibrotic responses even in the face of low PPAR-γ abundance. Currently approved PPAR-γ ligands such as rosiglitazone have been linked to side effects, including fluid retention, heart failure, and other cardiovascular events, raising questions regarding their safety [54]. Novel PPAR-γ agonists with selective activity and potentially improved safety are under active investigation [55].

In summary, these results reveal that PPAR-γ expression and activity are impaired in SSc, which is correlated with active TGF-ß signaling and fibrosis in lesional tissues. There exists a reciprocal relation between the expression of TGF-ß-activated genes and PPAR-γ-regulated genes in lesional tissue and in explanted SSc fibroblasts. PPAR-γ plays a physiologic role in tissue remodeling as a negative regulator of fibroblast activation and differentiation, which is highlighted by the link between loss of PPAR-γ and spontaneous emergence of fibrosis. Reduced PPAR-γ in SSc, resulting from its suppression by TGF-ß and related cytokines, hypoxia and other mechanisms might contribute to unchecked fibroblast activation and persistent fibrogenesis.

## Materials and Methods

### Ethics statement

The protocols for human tissue collection were approved by the Institutional Review Boards for Human Studies at Northwestern University and University of Pittsburgh. A written informed consent was obtained from all participants.

### Cell cultures

Primary cultures of dermal fibroblasts were established by explantation from biopsies of neonatal foreskins, or from affected dorsal forearm of seven patients with SSc and four healthy controls [3], [56]. Only patients with diffuse cutaneous SSc (dcSSc) who were not receiving treatment with corticosteroids were studied. Primary cell cultures were also established from the dorsal skin of newborn Smad3-null (Smad3^–/–^) mice or wildtype (Smad3^+/+^) littermates [30]. Unless otherwise indicated, all fibroblasts were maintained at 37°C in an atmosphere of 5% CO_2_ in Eagle's Minimum Essential Medium (EMEM) or Dulbecco's Modified Eagle's Medium (DMEM) supplemented with 10% fetal bovine serum (FBS), 1% vitamins, 1% penicillin/streptomycin, and 2 mM L-glutamine (all from BioWhittaker, Walkersville, MD), and studied between passages 4 and 8. When fibroblasts reached early confluence, fresh media containing TGF-ß (Genzyme, Framingham, MA) were added to the cultures. In selected experiments, cultures were pretreated with 15d-PGJ_2_ (BioMol, Plymouth Meeting, PA), troglitazone (GlaxoSmithKline, King of Prussia, PA), rosiglitazone (Cayman Chemical, Ann Arbor, MI), interleukin-4 (IL-4) or platelet-derived growth factor (PDGF) (both from Sigma, St. Louis, MO) for indicated periods. In other experiments, the protein kinase inhibitors U0126 (Cell Signaling Technology, Beverly, MA), SB431542 or SB203580 (both from Sigma) at 10 µM, or SP600125 (Sigma) at 20 µM, were added to the cultures 60 min prior to TGF-ß. In experiments focusing on profibrotic responses in fibroblasts, TGF-β at 10 ng/ml, a concentration shown to be maximally effective and non-toxic in prior studies, was used. Cell viability was determined by Trypan blue dye exclusion.

Human peripheral blood macrophages were isolated from buffy coats from healthy donors (Lifesource, Glenview, IL) by Histopaque (Sigma) gradient centrifugation followed by countercurrent centrifugal elutriation (JE-6B, Beckman Coulter, Palo Alto, CA). Isolated monocytes were ≥90% pure as determined by morphology, non-specific esterase staining and CD14 expression by flow cytometry. Following adherence to plates for 60 min, monocytes differentiated into macrophages for seven days in Roswell Park Memorial Institute (RPMI) media containing 20% heat-inactivated FBS, L-glutamine, and penicillin and streptomycin, and used in experiments.

### Adipogenic differentiation

Human subcutaneous preadipocytes (Zen-Bio Inc, Research Triangle Park, NC) or low-passage adult dermal fibroblasts were induced to undergo in vitro adipogenic differentiation as previously described [57]. Briefly, when cells reached confluence, cultures were incubated in DM-2 adipogenic induction media for three days, followed by AM-1 adipogenic maintenance media for a further three days (both from Zen-Bio). This cycle was then repeated three times. Following the final DM-AM cycle, incubation in AM was continued for up to a further 28 days prior to harvesting.

### Northern analysis and real-time quantitative PCR

Total RNA was isolated from confluent fibroblasts using TRIzol Reagent (Invitrogen, Carlsbad, CA), and levels of mRNA were determined by Northern analysis or real-time quantitative PCR (qPCR). Northern analysis was performed using α-^32^P-labeled human PPAR-γ 1 cDNA probes [13]. Following washing of the nitrocellulose membranes, the RNA-cDNA hybrids were visualized by autoradiography. For real-time quantitative qPCR, reverse transcription were performed using SuperScript First-strand synthesis system (Invitrogen) according to the manufacturer's protocol. For real-time qPCR, reactions were performed on ABI-Prism 7300 sequence detection PCR machine (Applied Biosystem, Forster City, CA) according to the manufacturer's protocol [58]. The primers used for qPCR are listed in Table 2. Relative mRNA expression levels were normalized with ß-actin mRNA or 18S RNA levels and determined by calculating ΔΔC_t_.

**Table 2:** Primers used for real-time quantitative PCR.

Gene		primers 5′–3′
human Col1A1	Forward	CCAGAAGAACTGGTACATCAG
	Reverse	CGCCATACTCGAACTGGAAT
human FABP4	Forward	GGAAAATCAACCACCATAAAG
	Reverse	GGAAGTGACGCCTTTCATGAC
human PPAR-γ1	Forward	GTCAAACGAGAGTCAGCCTTTAACG
	Reverse	CCACGGAGCTGATCCCAA
human ß-actin	Forward	AATGTCGCGGAGGACTTTGAT
	Reverse	AGGATGGCAAGGGACTTCCTG
mouse PPAR-γ1	Forward	GAGTGTGACGACAAGATTTG
	Reverse	GGTGGGCCAGAATGGCATCT
mouse 18S rRNA	forward	TTCGAACGTCTGCCCTATCA
	reverse	ATGGTAGGCACGGCGACTA

### Plasmids and adenoviruses

The PPAR-γ–luc plasmid contains 2.7 kb of the 5′ flanking region of the human PPAR-γ gene promoter linked to the luciferase reporter gene [59], [60]. The PPRE-luc plasmid contains 3 tandem copies of the PPAR response element (PPRE) sequence of the acyl-coenzyme A oxidase gene linked to thymidine kinase and luciferase genes [61]. A Smad3 expression vector has been previously described [62]. Replication-incompetent adenoviral vectors expressing constitutively active human TßRI (Ad-TßRI^ca^), L45 loop-mutated TßRI that lacks Smad binding ability (Ad-TßRI^L45^), and control adenovirus (Ad-null) were from Vesa Kaartinen [63].

### Transient transfection assays and adenovirus infections

Fibroblasts (10^5^ cells/well) were seeded in 12-well plates, and at early confluence, transiently cotransfected with reporter constructs and expression vectors or appropriate empty vectors using Superfect Reagent (Qiagen, Valencia, CA), or infected with adenoviruses. Following incubation with TGF-ß for 24 h, cultures were harvested and whole cell lysates were assayed for their luciferase activities [13]. The pRL-TK Renilla luciferase (pRL-TK-Luc) plasmid was used in each transfection experiment as an internal control. Experiments were performed in triplicate.

### Western analysis

At the end of the incubation periods, cultures were harvested, whole cell lysates or nuclear or cytosolic fractions were prepared, and aliquots containing equal amounts of proteins (10–15 µg) were subjected to Western analysis [13]. Membranes were immunobloted with primary antibodies to human PPAR-γ (1∶250 dilution) [64], Smad1/2/3, actin, TßRI, Smad7 (1∶200 dilution, all from Santa Cruz Biotechnology, Santa Cruz, CA), Smad3 (1∶250 dilution; Zymed), phospho-Smad2 (1∶1000 dilution; Cell Signaling Technology, Danvers, MA) or Type I collagen (1∶400 dilution; Southern Biotechnology, Birmingham, AL). Membranes were then washed and incubated with appropriate secondary antibodies for 45 min, and antigen–antibody complexes were visualized by chemiluminescence (Pierce Biotechnology, Rockford, IL) according to the manufacturer's instructions. The results were normalized against the intensity of the ß-actin in each sample.

### Immunocytochemistry

Fibroblasts were seeded on cover slides in a 6-well chamber and incubated in serum-free EMEM containing 0.1% BSA, TGF-ß (10 ng/ml) and rosiglitazone (10 µM). At the end of the incubation periods, cells were fixed and processed for immunocytochemistry [65]. The slides were incubated with primary antibodies against PPAR-γ or α-smooth muscle actin for 120 min, followed by Alexa® 488-conjugated chicken-anti-rabbit IgG or chicken-anti-mouse IgG (Invitrogen) for 60 min. Nuclei were identified with 4′-6-Diamidino-2-phenylindole (DAPI). Non-immunized IgG was used in each experiment as a negative control. Following stringent washing, slides were examined using Zeiss UV Meta 510 confocal microscope (Carl Zeiss, Jena, Germany). Each experiment was repeated at least three times with consistent results.

### Immunohistochemistry

Lung tissues were obtained from five patients with SSc-associated pulmonary fibrosis who were undergoing lung transplant surgery and from unused normal lungs from five donors. Sections (5 µm) were deparaffinized, rehydrated and immersed in TBS-T buffer (Tris-buffered saline −0.1%Tween 20), treated with target retrieval solution (DAKO, Carpinteria, CA) at 95°C for 10 min and incubated with primary antibody against PPAR-γ (1∶100 dilution, Santa Cruz). Bound antibodies were detected using secondary antibodies and System-HRP (DAB) (Dako, Glostrup, Denmark). Substitution of the primary antibody with isotype-matched irrelevant IgG served as negative controls. Sections were counterstained with hematoxylin, mounted with Permount (Fisher Scientific, Pittsburgh, PA) and viewed under an Axioskop microscope (Carl Zeiss). Pictures were taken and modified by Nuance Multiple Spectra CCD with Nuance 2.10 software. To quantify PPAR-γ expression, cells in five microscopic fields in each sample were scored as clearly immunopositive or negative for PPAR-γ by two independent examiners, and the ratio of positive cells to total cells in each field was calculated.

### DNA microarray analysis

The generation and analysis of the microarray dataset using skin biopsies from patients with SSc and normal controls was described previously [25]. The results of microarray analysis were ordered according to their “TGF-ß-responsive gene signature” expression [29]. To interrogate the expression of PPAR-γ target genes in the dataset, 87 genes known from the literature to be regulated by PPAR-γ were extracted from the microarray data. Those genes showing statistically significant differences in expression between “TGF-ß activated” and “TGF-ß not activated” subsets were selected for examination.

### Statistical analysis

The data are presented as means ± SD of multiple determinations. The significance of differences between experimental and control groups was determined by Student's *t*-test using GraphPad t-test calculator. A value of p<0.05 was considered statistically significant. For analysis of microarray data, Significance Analysis of Microarrays (SAM) using the Student's t-test function and data randomization to establish a False Discovery Rate (FDR) was used. Forty-one genes were selected with an FDR (q-value) of ≤0.78%.
